# The Role of Social Capital in Predicting Tourists’ Waste Sorting Intentions in Rural Destinations: Extending the Theory of Planned Behavior

**DOI:** 10.3390/ijerph191912789

**Published:** 2022-10-06

**Authors:** Jian Cao, Hongliang Qiu, Alastair M. Morrison, Wei Wei

**Affiliations:** 1 School of Tourism and Foreign Languages, Tourism College of Zhejiang, Hangzhou 311231, China; 2 School of Business Administration, Tourism College of Zhejiang, Hangzhou 311231, China; 3 Zhejiang Academy of Culture & Tourism Development, Hangzhou 311231, China; 4 School of Management and Marketing, Greenwich Business School, University of Greenwich, Old Royal Naval College, Park Row, London SE10 9LS, UK; 5 Department of Hospitality Services, Rosen College of Hospitality Management, University of Central Florida, Orlando, FL 32819, USA

**Keywords:** theory of planned behavior, social capital, tourists’ waste sorting intentions, rural tourism destination, rural tourism

## Abstract

Improper waste disposal of tourists has detrimental impacts on the environment, economy, and people in rural destinations. Separating at the source is an effective means to mitigate these adverse impacts on rural destinations. Hence, identifying factors influencing tourists’ waste sorting intentions in rural destinations is critical to the sustainability of rural tourism and rural land. However, few studies focus on tourists’ waste sorting intentions. Drawing on the theory of planned behavior (TPB) and social capital, this research examined the determinants of tourists’ waste sorting intentions in rural destinations. A total of 395 valid questionnaires were collected from a rural destination in Huzhou, China. The results indicated that: (1) all TPB variables, i.e., attitude toward the behavior, subjective norms, and perceived behavioral control, positively and directly affect tourists’ waste sorting intentions; (2) interpersonal trust directly and positively influences tourists’ waste sorting intentions; (3) subjective norms, perceived behavioral control, interpersonal trust, and emotional bonding indirectly influence tourists’ waste sorting intentions through the mediation of attitude toward the behavior; (4) emotional bonding does not directly affect tourists’ waste sorting intentions, but the link is established through the mediation of attitude toward the behavior. This research expands the body of knowledge by integrating individuals’ psychological elements with their social contexts. The findings offer some theoretical and managerial implications for understanding how tourists’ social contexts facilitate tourists’ waste sorting intentions.

## 1. Introduction

The growth of a tourism destination can have profound unfavorable impacts on the destination environment [1]. Rural destinations are not an exception. The COVID-19 pandemic has caused an immeasurable impact on the global tourism industry, but rural tourism destinations were relatively less affected and even experienced an increase in visitation due to better ecology and unique landscapes [2,3]. This resulted in a substantial increase in waste generated by tourists [4]. Statistics show that the tourism sector generates an approximate annual amount of 35 million tons of waste worldwide [5]. Improper waste disposal can have major and long-term effects on the natural environment and rural land [6]. Rural land involving diverse ecosystems is an essential component of rural tourism that potentially accelerates the growth of rural tourism [7]. Inadequate waste management will eventually cause soil contamination, water pollution, vegetation alteration, and ecosystem disruption [8]. These negative repercussions have raised the concern of both the public and researchers [9]. To mitigate these adverse impacts, one of the crucial and most effective solutions is waste sorting [10]. Particularly, effective waste sorting at rural destinations can dramatically improve environmental quality by maximizing the use of waste resources and decreasing waste disposal [11]. The success of waste sorting in tourism destinations heavily depends on tourists’ awareness and behavior. Therefore, it is of great importance to explore the determinants of tourists’ waste sorting behavior as a specific pro-environmental behavior.

A considerable amount of tourism literature has examined the reasons behind pro-environmental behaviors and intentions [12,13,14,15,16]. Tourists’ waste sorting behavior as a specific pro-environmental behavior is critical to the sustainable use of rural tourism and rural land [11]. Yet, it has not received enough attention from researchers. Regarding waste sorting, most prior studies investigate household waste sorting behavior and resident intention [17,18,19,20,21]. Unlike residents living in a daily environment, the anonymous nature of tourists’ identity during travel weakens moral obligations at destinations, which leads to distinct differences in their behaviors and intentions. Further, there is a dearth of literature on this topic in rural tourism. Compared with urban destinations, there are insufficient waste sorting facilities and a lack of waste sorting regulations in rural destinations. Nevertheless, the COVID-19 pandemic somewhat elevates the position of rural destinations [2,3]. Considering the importance and potential contributions of waste sorting to rural tourism destinations and rural land management, there is an urgent need to conduct a thorough investigation of the determinants of tourists’ waste sorting intentions in a rural tourism context.

Pro-environmental behavior refers to the broad actions of tourists in minimizing and preventing damage to the environment in destinations [22], either as a single-dimensional [23,24,25,26] or a multi-dimensional concept [27]. Several theoretical models have been applied to explain the formation of tourists’ pro-environmental behaviors and intentions, such as the theory of planned behavior (TPB), the stimulus-organism-response (SOR) model, the norm activation (NAM) model, and the cognition-affect-behavior (CAB) model [28,29,30]. Among these theoretical frameworks, the TPB is one of the most prominent rational-choice frameworks in understanding pro-environmental behavioral intentions [31]. The effectiveness of the TPB has been well established, and it mainly reflects individuals’ rational considerations of the cost–benefit balance for predicting pro-environmental behavioral intentions, including tourists’ waste sorting intentions [28]. Researchers have also noted that the TPB allows additional variables or notions to extend the model [32,33]. The fact that individuals also retain social traits has somewhat been ignored by the TPB [34]. We are not simply independent beings; instead, we are connected to and influenced by others and groups. Tourism researchers acknowledge that the social networks we are living in facilitate the formation of our cognition, attitudes, and behavior [35,36]. As such, social interactional aspects, such as interpersonal relations, social norms, and trust, should also be considered in the research of pro-environmental behavioral intentions. 

In response to this gap, social capital, which emphasizes the functions of social networks and interaction, is integrated into the TPB as a supplement in this research. Social capital is described as features of social organizations (including trust, norms, and networks) that can boost the efficiency of society by encouraging cooperative actions [37]. Social capital is a measure of how people interact with one another and how these interactions yield benefits for individuals and society [38]. Empirical studies of individual behavioral intentions and behaviors in various settings have validated the efficacy of social capital [39,40,41,42]. Recently, a few researchers have examined household waste sorting behavior and intention with social capital [43,44], while more studies in the research of pro-environmental behaviors and intentions can be found to support the effective application of social capital [35,36,45,46]. Due to the complexity of individual behavior, tourists’ psychological factors alone cannot fully capture their waste sorting intentions. Their decisions also heavily depend on social environments and interactions with other tourists and destinations. Yet, extant studies have not explained waste sorting intention by combining tourists’ psychological and social interactive factors. This research extends the TPB model with social capital to reveal both psychological and social interactive factors that influence tourists’ waste sorting intentions. 

Overall, to fill the above-mentioned gaps, this study aims to: (1) integrate social capital into the TPB model for understanding tourists’ waste sorting behavior; (2) identify the main determinants of tourists’ waste sorting intentions; and (3) test the mediating role of attitude toward the behavior from the perspective of tourists. This research enriches our understanding of tourists’ waste sorting intentions from a social interactive perspective. The extended TPB model with social capital offers new insights into travelers’ decisions to sort waste in rural destinations. Practically, it may offer guidelines for destination managers to utilize tourists’ social networks in promoting waste sorting intentions.

## 2. Literature Review and Hypotheses Development

### 2.1. Tourists’ Waste Sorting Intentions

Waste sorting is defined as people’s engagement in the appropriate separation of waste items into various categories for beneficial waste management, such as reuse or recycling [44,47]. Appropriate waste sorting at the source makes it easier to collect, transport and dispose of the waste, which contributes significantly to environmental protection. Likewise, tourists’ waste sorting behavior in destinations effectively reduces the negative effects of tourism waste on the sustainable development of rural destinations and rural land. To this end, tourists’ waste sorting behavior can be understood as a specific type of pro-environmental behavior, which refers to behaviors of lessening and preventing the environmental destruction of destinations [15,48]. The importance of pro-environmental behavior has also been widely recognized in tourism research [49]. To date, however, the existing literature on waste sorting has fallen short of examining waste sorting intention as a specific pro-environmental behavior in rural destination settings.

### 2.2. The Theory of Planned Behavior (TPB)

The TPB is a derivation of the theory of reasoned action (TRA), which was developed by Ajzen to predict an individual’s decision-making process [32]. According to the TRA, the most direct predictor of actual behavior is one’s intention to perform the behavior, while behavioral intention is a function of two volitional determinants, i.e., subjective norms and attitude toward the behavior [33]. To increase the predictability of the TRA, Ajzen extended the theory by adding a non-volitional construct (perceived behavioral control) into the model, forming the TPB model [32]. According to the TPB, more positive attitudes and subjective norms for a given behavior, as well as a stronger feeling of behavioral control, lead to a more intense intention of performing the behavior. 

As one of the most influential theories for understanding an individual’s behaviors, the TPB has been successfully utilized to explain pro-environmental behaviors and intentions in tourism and hospitality, such as green purchasing intention [50], willingness to pay more [51], scuba divers’ underwater behavior [52], and binning behavior [53]. A few studies, though not in tourism research, particularly focus on explaining one specific type of pro-environmental behavior (i.e., waste sorting) within the TPB theoretical framework [54,55,56,57]. These empirical studies have validated the efficacy of the TPB in predicting pro-environmental behaviors and intentions, including waste sorting intention. Considering the lack of research on waste sorting intention from the tourist perspective, this research employed the TPB as the fundamental theoretical framework for understanding waste sorting intention in rural destinations.

### 2.3. Impact of the TPB Variables on Tourists’ Waste Sorting Intentions

Attitude toward the behavior refers to the degree of an individual’s favorable or unfavorable judgment or assessment of the behavior in question [32]. It is associated with one’s assessment of the possible outcomes of a specific behavior and the corresponding favorable or unfavorable judgment about the outcomes. In tourism, when visitors believe that pro-environmental behaviors, such as waste sorting, will bring positive or valuable outcomes, they will have stronger intentions to perform this behavior. This positive association has been confirmed by extensive research on pro-environmental behaviors and intentions [58,59]. The previous waste management literature also reports the positive relationship between attitude toward behavior and behavioral intentions [4,60], including waste sorting intention. For example, in examining residents’ waste sorting intentions, attitude toward the behavior was shown to be effective in predicting household waste sorting intention [61]. Thus, this research proposed the following hypothesis:

**Hypothesis** **1** (**H1**)**.** *Attitude toward the behavior is directly and positively related to tourists’ waste sorting intentions.*

Subjective norms are described as the social pressure on an individual to engage in a behavior [32]. The social pressure from a given reference group (such as relatives, peers, or a community at large) affects people’s willingness to adopt subjective norms due to the attribution of being socially acceptable and appropriate [62]. Otherwise, they may be excluded from this group or community. This is the main motivation for people fulfilling the norms [63]. As the TPB assumes, subjective norms play a substantial role in predicting one’s intentions [32]. The connection between subjective norms and pro-environmental behavioral intentions has been well established in the tourism literature. For example, in investigating outbound tourists’ pro-environmental behavioral intentions, it has been found that subjective norms play a significant role in intentions to perform pro-environmental behavior [64]. When examining waste sorting behavior, previous studies indicate that subjective norms significantly and directly affect people’s behavior [18,65]. 

Additionally, cognitive dissonance theory assumes that an individual will consciously change attitudes to comply with group norms and stay consistent with the views and behaviors of important group members [66]. This may offer insights into the links between subjective norms and attitude toward the behavior. The extant literature also suggests that these two variables are not mutually independent; to be specific, there is a positive connection between them [59]. Findings of other empirical studies echo this view, i.e., subjective norms have a positive impact on attitude toward the behavior [28,67]. Based on the above discussion, this research suggested the following hypotheses:

**Hypothesis** **2** (**H2**)**.** *Subjective norms are directly and positively related to tourists’ waste sorting intentions.*

**Hypothesis** **3** (**H3**)**.** *Subjective norms are directly and positively related to attitude toward the behavior.*

Perceived behavioral control is understood as people’s perceptions of their ability to perform a certain behavior [32]. Perceived behavioral control illustrates people’s feelings of ease or difficulty in carrying out a specific behavior based on the resources or abilities they acquire [32]. If an individual perceives a high level of control over the behavior, this perception, in turn, enforces their engagement in that behavior [28]. The findings of prior research indicate a significant and positive link between perceived behavioral control and one’s waste sorting intention [20]. More evidence supporting this link can be found in the literature on tourists’ pro-environmental behavior [31,52]. In addition, previous studies indicate that perceived behavioral control is also correlated with attitude toward the behavior [68]. In a comparative study of tourists’ pro-environmental behavioral intentions in nature-based destinations, scholars found that perceived behavioral control was positively linked with attitude toward pro-environmental behavioral intentions [28]. Thus, it was hypothesized that:

**Hypothesis** **4** (**H4**)**.** *Perceived behavioral control is directly and positively related to tourists’ waste sorting intentions.*

**Hypothesis** **5** (**H5**)**.** *Perceived behavioral control is directly and positively related to attitude toward the behavior.*

### 2.4. Social Capital 

Despite its value in explaining individual behavior, the TPB also receives criticism since it only emphasizes individual cognitive factors [69], which restricts its explanatory power. Depending on various settings, the TPB needs to be extended with other concepts or variables to improve its predictivity [32]. Since waste sorting intention as a specific pro-environmental behavioral intention is altruistic and collectivistic in nature, the social contextual factors should be considered for a holistic understanding of the decision-making process. In this sense, social capital, which focuses on the impact of an individual’s social networks and interpersonal relationships, is suitable for extending the TPB model for predicting tourists’ waste sorting intentions. 

As a century-old concept, social capital did not receive significant attention from academia until the 1980s [45]. It is generally concurred that Pierre Bourdieu, who introduced the economic notion of “capital” into social studies, laid the groundwork for contemporary research on social capital [70]. Bourdieu stressed the importance of social networks from the individual perspective [71]. Colman described trust, social norms, and networks as the constituents of social capital, making a tremendous contribution to the theorization of social capital [72]. It was not until Putnam’s investigation of social capital’s role in the collaboration of social groups that the importance of social capital was well acknowledged [37,73]. 

Social capital refers to the connectedness of interpersonal relationships linked to a given population and “soft resources” formed in social interactions for the owners to obtain other resources [37,74,75]. The rationale behind the concept is that social interactive factors such as interpersonal relationships, social trust, and norms embedded in the social networks foster one’s attitude, cognition, and behavior and bring benefits to the individual and groups [38,71,72,75]. Empirical studies in various settings have validated the efficacy of social capital in explaining individual pro-environmental behaviors [35,45,76,77,78], as well as tourists’ pro-environmental behavior [36]. Regarding waste sorting behavior, Wang et al. found that different dimensions of social capital facilitate individual participation in waste sorting, mainly through the pro-environmental norms in social networks [44]. 

Social capital has been defined and classified differently by researchers in various fields [79,80,81]. These definitions and classifications of social capital contribute to a better understanding of its application in various domains. However, most of these efforts try to argue for a particular view of social capital at the cost of others [38]. The complexity of our daily lives determines the diversity of our social interactions. The notion of social capital should be based on various facets of people’s social lives. Based on three types of social capital usage, Brunie (2009) proposed a three-dimensional concept of social capital, which encompasses relational, collective, and generalized social capital. These three aspects of social capital are closely related to people’s participation in altruistic and collective behaviors, such as waste sorting. Referring to Li and Wu, emotional bonding, in-group norms, and interpersonal trust represent the different dimensions of Brunie’s conceptualization of social capital, respectively, i.e., relational, collective, and generalized social capital [36]. 

It is worth noting that collective social capital is considered as a collective resource that enables the collaboration among small groups for mutual benefits [38]. Unlike the intentional mobilization of a specific individual’s relations, it emphasizes the quality or intensity of the social interactions among members within a given group. While voluntary cooperation comes from norms of reciprocity and social networks, which discourage opportunistic acts of group members [82,83,84]. Viewed from the individual perspective, this notion is akin to the subjective norms in TPB, which also assumes that social pressure from a given social network affects the behavior of the actors in the social network. On this level, the collective social capital (or in-group norms) is equivalent to social norms in TPB [36]. Thus, this research considers them as the same notion and integrates collective social capital into the subjective norms of TPB in the theoretical framework. Since the impact of subjective norms on tourists’ waste sorting intentions have been discussed above, the impact of social capital on waste sorting intention will be expounded from the relational and generalized approaches, i.e., emotional bonding and interpersonal trust. 

### 2.5. Impact of Different Social Capital Dimensions on Tourists’ Waste Sorting Intentions

Relational social capital is approached from the mutual relationships between individuals, which facilitates people to obtain helpful information, transaction opportunities, or influence [38]. As such, relational social capital can be understood as the outcome of deliberate investment in social relationships, which gives actors different access to resources and benefits (such as finding better jobs, seeking emotional support, or getting a promotion). Previous studies show that emotional bonding is the main channel for accessing and mobilizing valuable resources [85]. The social exchange theory proposes that the action of one party triggers similar reciprocal actions of the other [86]. When a strong emotional bonding exists in this social interaction, the parties involved are more inclined to exchange each other’s unique resources for mutual benefits [87]. In tourism, this rationale is also applicable in the relationship between the tourist and the destinations [36]. A satisfactory tourism experience connects visitors and destinations emotionally. In turn, this emotional bonding prompts visitors to perform pro-environmental behaviors at destinations in return. This emotional bonding between visitors and destinations is defined as “place attachment” in environmental psychology [88]. The significance of place attachment in promoting pro-environmental behavior has been verified in a considerable number of studies [89,90,91]. 

Moreover, in understanding the formation of individual behavior, many social psychological theories share the view that a causal relationship exists between attitudes and behavior or behavioral intentions [92]. One such theory is the expectancy value model, which holds that one’s beliefs about an object and assessments of these beliefs constitute an attitude [93]. Along this line, emotional bonding with a place can be described as beliefs about a place [94], which are the foundations of attitudes [95]. In examining the role of tourists’ emotional bonding toward a specific place in their decision-making process, scholars have found that the sense of bonding significantly affects attitudes [33,36]. Accordingly, this research hypothesizes that:

**Hypothesis** **6** (**H6**)**.** *Emotional bonding is directly and positively related to tourists’ waste sorting intentions.*

**Hypothesis** **7** (**H7**)**.** *Emotional bonding is directly and positively related to attitude toward the behavior.*

Generalized social capital is described as the values and attitudes that shape interpersonal interactions and enable individuals to trust, cooperate, and sympathize with others publicly [38]. Interpersonal trust is the cornerstone of generalized social capital [38]. In contrast to the profound trust within a clearly defined group in collective social capital, generalized trust pertains to a much broader community. It is understood as a loose sense of trust that encourages collaboration among unfamiliar people for good causes, such as pro-environmental behaviors. Regarding environmental protection, however, it is suggested that there is a contradiction between individuals’ own interests and collective interests [96]. As an altruistic and collective behavior, pro-environmental behavior such as waste sorting requires visitors to contribute extra effort (such as time and money) for a clean and sustainable environment. Without trust among unfamiliar tourists, they may feel reluctant to do so for fear of free riders. The association between interpersonal trust and waste sorting behavior has been validated in prior research [44]. In the literature on pro-environmental behavior, more evidence can be found to support the link between interpersonal trust and tourists’ pro-environmental behavior [27,36,97]. 

Additionally, studies in diverse settings have highlighted how one’s attitudes may be influenced by trust [69,98,99,100]. In tourism, for example, scholars investigating the role of trust on travelers’ travel decisions during and after the COVID-19 pandemic observe that people’s attitudes toward travel were affected by trust among travelers. That is, when they trust other visitors are following health precautions during travel, they will have more positive attitudes toward traveling and are more likely to decide to take action during and after the pandemic [101]. This nexus has also been confirmed in the study of behavior toward the adoption of self-service technology, in which trust is found to have a more significant impact on visitors’ attitudes than subjective norms [102]. The extant research indicates that generalized trust may positively affect tourists’ attitudes toward the behavior. Based on this backdrop, this research put forward the following hypotheses:

**Hypothesis** **8** (**H8**)**.** *Interpersonal trust is directly and positively related to tourists’ waste sorting intentions.*

**Hypothesis** **9** (**H9**)**.** *Interpersonal trust is directly and positively related to attitude toward the behavior.*

**Figure 1:**
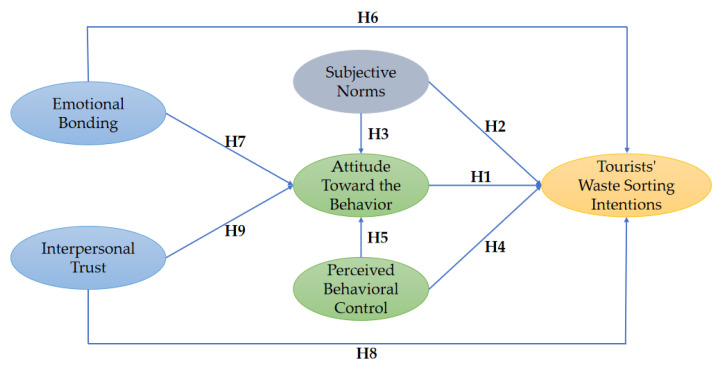
Integrated conceptual framework for predicting tourists’ waste sorting intentions in rural tourism. Note: The subjective norms in the TPB model are equivalent to the in-group norms in social capital.

Based on the above review of the literature, the integrated conceptual framework for this research was proposed as follows (Figure 1).

## 3. Method 

### 3.1. Measurement 

Each construct was measured by multiple previously validated items. Specific adjustments for the item scales were made according to the research setting (see Table 1 for detailed measurement). All items were assessed on five-point Likert scales, ranging from 1 (strongly disagree) to 5 (strongly agree). It is worth noting that one of the indicators (i.e., *I am filled with doubts that other visitors would sort the waste in this destination*) was reverse coded and required adjustment and recalculation in the subsequent analysis.

### 3.2. Pretest of Measurements 

The English–Chinese translation and back-translation approaches were adopted to ensure the translation accuracy and item applicability [33]. Two tourism researchers and two destination practitioners were invited to conduct a pretest for accessing content/face validity. Some 65 Chinese tourists who had traveled to the research site participated in the pilot study. The preliminary results saw an acceptable reliability (Cronbach’s alpha > 0.70) and validity (standard factor loadings > 0.50) for the measurement scales at this stage [104,105]. 

### 3.3. Data Collection

**Figure 2:**
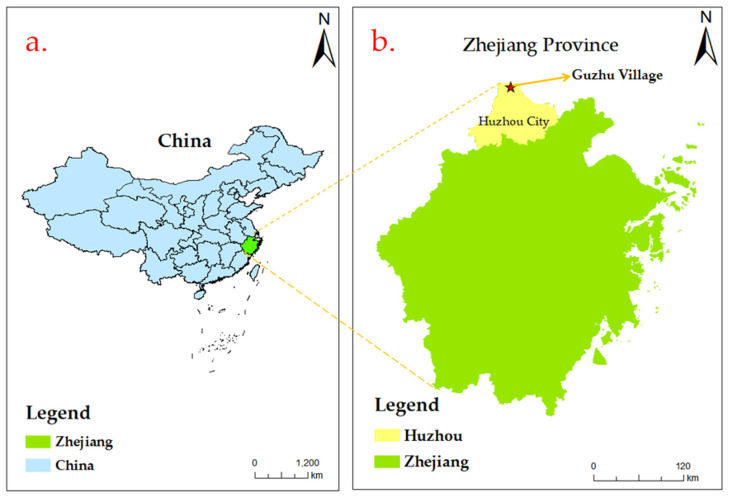
(**a**) Geographical location of Zhejiang Province in the People’s Republic of China; (**b**) Geographical location of Guzhu Village in City Huzhou, Zhejiang Province.

**Figure 3:**
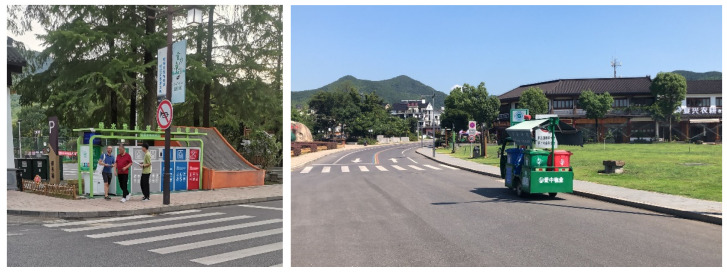
The photograph on the left shows the researchers performing the field survey next to the smart waste sorting facility; while the right one shows the waste collecting vehicle equipped with a loudspeaker broadcasting how to sort waste repeatedly when it is at work.

The data were collected from a village named Guzhu in Huzhou City, Zhejiang Province, China. Guzhu Village is well-known for agritainment, with over 500 farm stays. The annual tourist arrivals are more than four million. It has been rewarded with several national-level and provincial-level titles, such as national forest village, a national pilot village for agritourism, and provincial pilot village for wellness tourism. Located at the junction of three provinces (Zhejiang, Jiangsu, and Anhui), it transformed from an impoverished village into a famous destination for agritainment over the past two decades [106]. Accordingly, this research considers this destination as a qualified site for the field survey of rural tourism (See Figure 2 for the geographical location of Guzhu Village and Figure 3 for photos of researchers conducting the field survey). 

Domestic tourists accounted for the majority during the fieldwork mainly because of the impacts of the COVID-19 pandemic and travel restrictions on international arrivals [30]. An on-site survey was conducted by three research teams of one trained research assistant and one researcher in early July 2022. The survey adopted the convenience sampling method. After verbal consent and detailed instruction, respondents were invited to participate in the survey and offered free gifts such as masks and folding fans. Those who refused or were unqualified as domestic tourists were replaced by the next available respondent. Following the above procedure, some 440 questionnaires were distributed, of which 395 questionnaires were valid, with an 89.8% effective response rate. The data presented a relatively balanced gender ratio (47.1% of males and 52.9% of females). In terms of age of the respondents, 25.6% of them were aged below 25; 32.2% between 25 and 44 years old; 23.5% 45–59 years old; and 18.7% 60 years old and above. As for the educational background, 13.9% of the participants only received middle school education or below; 42.0% attended high school or technical secondary school education; 44.1% received undergraduate degrees and above. The values of univariate skewness statistics (−0.701 to 0.676) and kurtosis statistics (−0.982 to 0.131) met the skewness and kurtosis criteria [107,108].

## 4. Results

### 4.1. Common Method Bias Test

In survey-based research, a common method bias (CMB) test is required, especially when the source of the received data is the same [109,110]. First, the factor analysis tool in SPSS was utilized to execute Harman’s single-factor test [111]. According to the test results, CMB was not an issue since no single factor explained over 50 percent of the covariance, with the first factor accounting for 36.086% of the total variance [112]. Second, confirmatory factor analysis was performed to examine if a common latent factor explained all of the variance. The proposed measurement model was preferable to the common factor model (Δχ2(15) = 5206.95, *p* < 0.001). Therefore, CMB was not a problem for the present study.

### 4.2. Measurement Model Analysis 

The reliability and validity of the constructs, and the measurement model fit, were evaluated with confirmatory factor analysis before assessing the proposed hypotheses [29]. According to the model fit indices (χ^2^/df = 2.380, RMSEA = 0.059, RMR = 0.015, NFI = 0.947, CFI = 0.969, IFI = 0.969, GFI = 0.905, TLI = 0.963, SRMR = 0.0302), the measurement model fit the data well. As Table 2 presents, Cronbach’s alpha values spanned from 0.808 to 0.974 for each construct, suggesting acceptable internal reliability of the measurement model [113]. Additionally, two different construct validity measures were evaluated (convergent and discriminant validity). Table 2 shows that the composite reliability varied from 0.820 to 0.974. A satisfactory convergent validity was demonstrated by the standardized factor loadings, average variance extracted (AVE), and composite reliability of each construct [114,115]. The square root of each construct’s AVE was compared with the correlations between corresponding latent constructs to calculate the discriminant validity [114]. Table 3 presents clear evidence of discriminant validity. Thus, the reliability and validity of the measurement model were both established, which warranted further hypothesis testing of the structural model.

### 4.3. Examining Structural Model 

**Figure 4:**
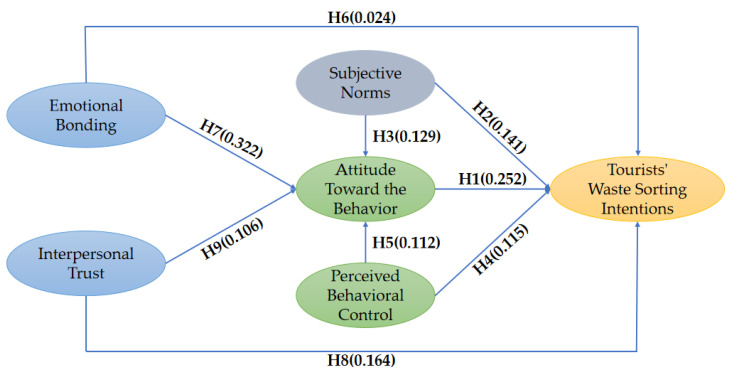
AMOS output results of the integrated structural model. Note: The subjective norms in the TPB model are equivalent to the in-group norms in social capital.

Structural equation modeling (SEM) was applied to examine the direct hypotheses. The fit indices implied that the structural model had a good fit (χ^2^/df = 2.380, RMSEA = 0.059, RMR = 0.015, NFI = 0.947, CFI = 0.969, IFI = 0.969, GFI = 0.905, TLI = 0.963, SRMR = 0.0302). The results showed that eight of the nine hypothesized direct relationships were supported (Table 4). There was no significant direct relationship between emotional bonding and tourists’ waste sorting intentions (β = 0.024, *p* > 0.05), meaning that H6 was not supported. Figure 4 presents the AMOS output results of the structural model. 

### 4.4. Mediating Effect Test

Various approaches have been applied to test hypotheses about mediation effects, such as the causal steps approach, the Sobel test, and the bootstrapping method. The causal steps approach proposed by Baron and Kenny [116] is the most widely-used approach to address this issue. However, this approach has two main limitations. On the one hand, simulation studies have shown that the causal steps method is one of the least powerful methods for testing mediation effects [117,118]. On the other hand, this method does not calculate the magnitude of the mediation effect [119] and cannot accommodate frameworks with inconsistent mediation [120]. The Sobel test is employed occasionally, but it is frequently used as a supplement to the causal steps approach rather than a replacement [121]. The Sobel test demands the premise that the sampling distribution of the indirect effect is normal. However, the sampling distribution of *ab* is often asymmetric and has nonzero skewness and kurtosis [122]. Bootstrapping with a confidence interval is considered to be preferable to the conventional Sobel test since bootstrapping can avoid a high Type I error rate owing to the violation of the normal distribution [123]. Recently, a considerable number of studies have applied the bootstrapping method to test mediation effects [13,15,30,33,107].

Accordingly, the bootstrapping method in AMOS was applied to test the mediating effect. The number of bootstrap samples was set to 5000 with bias-corrected and percentile confidence intervals at 95% [33]. For example, a significant specific mediating effect was observed for subjective norms on tourists’ waste sorting intentions via attitude toward the behavior (95% CI_bias-corrected_: [0.005, 0.072]; 95% CI_percentile_: [0.003, 0.068]) (Table 5). Likewise, the following specific indirect paths were supported: PBC→ATT→TWSI (95% CI_bias-corrected_: [0.005, 0.062]; 95% CI_percentile_: [0.002, 0.059]), EB→ATT→TWSI (95% CI_bias-corrected_: [0.043, 0.130]; 95% CI_percentile_: [0.041, 0.127]), and IT→ATT→TWSI (95% CI_bias-corrected_: [0.005, 0.056]; 95% CI_percentile_: [0.004, 0.023]).

### 4.5. Explanatory Power of the Conceptual Model

The explanatory power of this research’s conceptual model was analyzed by the *R*^2^ of the major endogenous variables. The *R*^2^ values were 0.25, 0.09, and 0.01 for the threshold of large, medium, and small effects, respectively [124]. Table 6 presents the results from the squared multiple correlations (SMC = R^2^) and implies that the theory of planned behavior (i.e., M0) explains 19.5% of the variance for tourists’ waste sorting intentions; the social capital model (i.e., M1), 15.3%; the integrated model (i.e., M2), a higher 22.0%. The findings implied that the integrated model had greater merit than the single model in terms of explanatory power. 

## 5. Conclusions and Implications 

### 5.1. Conclusions

Understanding the formation of tourists’ waste sorting intentions is of great significance to waste management of rural land and the sustainability of rural tourism destinations. The current research extended the TPB framework with social capital to investigate and verify the effects of individual rationality and social contexts on tourists’ waste sorting intentions. With the data collected from a rural tourism destination known for its efforts in environmental conservation, the empirical results indicated that most of the proposed research hypotheses were supported.

First, the results confirmed that tourists’ waste sorting intentions were positively affected by all three constructs of the TPB, namely, attitude toward the behavior, subjective norms, and perceived behavioral control. This is in line with the empirical findings of various studies using the TPB to examine how TPB variables directly influenced tourists’ pro-environmental behaviors and intentions [59,125]. It implies that visitors are more predisposed to sort waste in destinations if they have a more positive attitude toward waste sorting in conjunction with a stronger sense of subjective norms and perceived behavioral control. Accordingly, it confirms the plausibility of the TPB framework in predicting specific tourists’ pro-environmental behavioral intentions [33,126]. 

Second, the effect of social capital was examined from relational and generalized approaches, i.e., emotional bonding and interpersonal trust. As an interchangeable concept of subjective norms, the direct and positive connection between subjective norms and tourists’ waste sorting intentions in the TPB model confirmed the effect of collective social capital (i.e., in-group norms). The results indicated that interpersonal trust directly and positively influenced waste sorting intention. This is consistent with the results of prior research on tourists’ pro-environmental behavioral intentions [36,89,97], as well as in the study of household waste sorting [44]. It means that interpersonal trust and in-group norms embedded in people’s social networks also play essential roles in the formation of their pro-environmental behavioral intentions, which, in turn, reward individuals and groups [38,75]. Surprisingly, emotional bonding did not directly influence tourists’ waste sorting intentions but through the mediation of attitude toward the behavior. This is inconsistent with the findings of some previous research [36,89,97]. It is argued that this may be related to the cost and convenience of waste sorting in this rural destination. The mandatory waste sorting policy implemented in the village has significantly improved people’s awareness of sorting waste, either in urban communities or rural areas. Waste sorting facilities are very easy to find in rural destinations, which substantially increases the ease of waste sorting. As a result, tourists with a strong sense of bonding with the place may only need to reinforce their attitude toward the behavior to improve their waste sorting intentions [127]. Since social capital has rarely been applied to explain waste sorting intention in rural tourism, the findings further the understanding of its role in specific pro-environmental behavioral intentions in rural tourism.

Finally, the mediating role of attitude toward the behavior was tested in the extended TPB theoretical framework. The results demonstrated that there is a direct and positive link between emotional bonding, interpersonal trust, and attitude toward the behavior. Moreover, attitude toward the behavior also connected other TPB variables (i.e., subjective norms and perceived behavioral control) to behavioral intentions. This follows the cognitive dissonance theory and supports prior studies on the relationship between subjective norms, perceived behavioral control, and attitude toward the behavior [28]. Additionally, it also echoes the role of social capital in triggering general and specific pro-environmental behavior and intentions in the previous literature [35,78]. Thus, the present research showcased the role of attitude toward the behavior in facilitating tourists’ waste sorting intentions from both individual rationality and social interaction aspects. 

### 5.2. Theoretical Contributions 

Through integrating social interactional elements into the TPB model, the present research offers new insights into how social networks and people’s rationality work together to exert an influence on waste sorting intention in rural tourism. It provides the following theoretical implications for the sustainability of rural tourism destinations and rural land.

First, this current study employs the TPB to explain a specific tourists’ pro-environmental behavioral intention (i.e., waste sorting) in rural tourism destinations. It is generally agreed that the TPB is one of the most widely applied theories in predicting individual behaviors [31], including residents’ household pro-environmental behavioral intentions such as waste sorting intentions [55]. In the domain of specific tourist pro-environmental behavioral intentions, however, the TPB has rarely been applied to examine waste sorting intention. The finding of this study supports the direct links between the TPB variables (i.e., attitude toward the behavior, subjective norms, and perceived behavioral control) and tourists’ waste sorting intentions, which showcases the robustness of the TPB in predicting waste sorting intention in rural destinations. Accordingly, the present study sheds new light on the feasibility of the TPB in the research of waste sorting intention in rural tourism, extending the application of the TPB in the research of pro-environmental behavioral intentions. 

Second, social capital is integrated into this research to explain how social ties affect pro-environmental behavioral intentions. The integration of social capital offers a novel perspective to examine how tourists’ social relations contribute to specific pro-environmental behavioral intentions, such as waste sorting. Specifically, the finding reveals that in-group norms (i.e., subjective norms) and interpersonal trust are directly associated with waste sorting intention, while attitude toward the behavior mediates the indirect links between emotional bonding, in-group norms, interpersonal trust, and waste sorting intention. In the previous literature on waste sorting intention, the explanatory power of social capital has been confirmed in the household setting [44]. However, there is inadequate evidence of rural tourism. Hence, this study expands the application of social capital to the research on waste sorting intention in rural tourism.

Finally, this current study applies the TPB model, along with social capital, to understand waste sorting intention in rural tourism. The efficacy of either the TPB or social capital has been verified in explaining waste sorting intention [44,56]. However, to the researchers’ best knowledge, this research is one of the first attempts to examine the influence of cognitive factors (i.e., attitude toward the behavior, subjective norms, and perceived behavioral control) and social contextual factors (e.g., emotional bonding, interpersonal trust) on waste sorting intention in rural tourism destinations, which may advance the understanding of tourists’ adoption of these specific pro-environmental behavioral intentions in rural tourism. In this way, the present study might help explain waste sorting intention in rural tourism destinations by developing an extended TPB framework. The empirical results of this study have shown the merits of the integrated theoretical framework over either the TPB or social capital. 

### 5.3. Managerial Implications 

The findings of this study highlight the contribution of social capital in encouraging tourists to sort waste in rural tourism destinations, which provides several managerial implications for destination management. 

First, the effects of the TPB variables (i.e., attitude toward the behavior, subjective norms, and perceived behavioral control) on tourists’ waste sorting intentions have been highlighted in this research. Destination management departments should enhance tourists’ perception of their ability and contribution to the environment through publicity campaigns and waste sorting facilities. For example, smart waste collection monitoring technology is used at conspicuous places in the rural destination visited for the field survey. The monitor above the smart waste bins shows statistics about the waste sorted in a certain period, including the weight, proportion, and types of waste, as well as the record of each sorting behavior via the camera. Visitors can easily see their contribution to waste management at the destination and feel pressure if they have disposed of the waste randomly. Posters promoting waste sorting knowledge are almost everywhere in the destination. These efforts help improve the attitude toward waste sorting and boost visitor confidence in being able to sort waste. 

Second, the integrated theoretical framework of this research indicated that social contextual elements also affect behavioral intentions for the public good in addition to internal factors. Consequently, strategies are needed to influence various social interactional factors to trigger changes in intentions for waste sorting. For example, the official social network account of the destination is a helpful tool for interactions between visitors and the destination. Online events on how to sort waste can be organized to reinforce the interaction and increase visitors’ knowledge about waste sorting. Rewards or discounts can be offered to raise visitors’ interest in engagement and to share in their personal social networks to improve their bonding with the destination. 

Third, the results of this research underlined the significance of subjective norms (or in-group norms) in the formation of waste sorting intention. It means that attention should also be paid to important companions of visitors to the destinations, such as family members, coworkers, and friends. The social pressure from these important companions can exert considerable influence on behavioral intention. Hence, waste sorting awareness campaigns or themed parent–child activities can be held, in which some subjective norms can be highlighted to facilitate the promotion and advocacy of waste sorting in tourism destinations. 

Lastly, the effect of interpersonal trust should be emphasized in the practice of destination management. The results of this study identified the direct connection between interpersonal trust and waste sorting intention, along with the role of perceived behavioral control in explaining waste sorting intention. Thus, waste sorting initiatives at the destination need to reinforce the self-efficacy and group efficacy perceived by tourists. Messages should be sent to visitors via these initiatives so that everyone can sort the waste with little cost during travel while also contributing to destination sustainability. 

## 6. Limitations and Future Research Directions 

Despite contributions to the research on tourism sustainability, the present study had some limitations that may offer guidance for future research. First, the self-administered approach was employed in this study to capture tourists’ waste sorting intentions. However, the experimental method is increasingly popular among scholars [128,129,130]. Second, the cross-sectional data obtained for this research may not fully explain the causality within the extended TPB framework. Thus, longitudinal data may be needed to track changes in waste sorting intention over time. Third, data were collected from a single type of destination, i.e., the rural destination. Future research should consider including urban destinations for cross-validation to improve the applicability and reliability of the research framework. Finally, this research used the SEM method to examine the linear relationships between variables. Future researchers should consider the fuzzy-set qualitative comparative analysis because it focuses on the asymmetric relationships between variables, which can facilitate a better understanding of the non-linear effect [27]. 

## Figures and Tables

**Table 1:** Detailed measurements of all variables.

Construct	Item	Source
Attitude toward the behavior (ATT)	ATT1 During this trip, I thought waste sorting was a wise behavior.	[30]
	ATT2 During this trip, I thought waste sorting was a valuable behavior.	
	ATT3 During this trip, I thought waste sorting was a necessary behavior.	
	ATT4 During this trip, I thought waste sorting was a beneficial behavior.	
Subjective norms/ In-group norms (SN)	SN1 During this trip, those important to me thought I should sort waste.	[36]
	SN2 During this trip, those important to me expected me to sort waste.	
	SN3 During this trip, those important to me were delighted if I sorted waste.	
Perceived behavioral control (PBC)	PBC1 During this trip, whether or not I sorted waste was up to me.	[30]
	PBC2 During this trip, I was capable of sorting the waste.	
	PBC3 During this trip, I was confident that if I wanted, I could sort waste.	
Emotional bonding (EB)	EB1 I identify strongly with this destination.	[36]
	EB2 Visiting this destination says a lot about who I am.	
	EB3 I am very attached to this destination.	
	EB4 I feel visiting this destination is part of my life.	
	EB5 This destination means a lot to me.	
	EB6 I feel a strong sense of belonging to this destination.	
Interpersonal trust (IT)	IT1 I believe that most visitors sort waste in this destination.	[36]
	IT2 Regarding promoting waste sorting in this destination, I believe that my individual behavior is impactful because many others will contribute too.	
	IT3 I am filled with doubts that other visitors sort waste in this destination. (Reverse coded)	
Tourists’ waste sorting intentions (TWSI)	TWSI1 I intend to sort waste at this destination.	[103]
	TWSI2 I am willing to sort waste at this destination.	
	TWSI3 I am planning to sort waste at this destination.	

**Table 2:** Measurement model results.

Construct and Item	Std. Factor Loading	*t* Values	Composite Reliability	Average Variance Extracted	Alpha
ATT			0.954	0.838	0.953
ATT1	0.872	26.754			
ATT 2	0.932	32.032			
ATT 3	0.945	33.454			
ATT 4	0.912	—			
SN			0.924	0.803	0.922
SN1	0.828	24.043			
SN2	0.909	29.589			
SN3	0.947	—			
PBC			0.912	0.776	0.910
PBC1	0.824	21.381			
PBC2	0.932	25.283			
PBC3	0.884	—			
EB			0.955	0.781	0.955
EB1	0.869	25.931			
EB2	0.848	24.532			
EB3	0.921	29.999			
EB4	0.872	26.132			
EB5	0.886	27.16			
EB6	0.903	—			
IT			0.820	0.608	0.808
IT1	0.892	11.977			
IT2	0.803	12.111			
IT3 (Reverse coded)	0.619	—			
TWSI			0.974	0.926	0.974
TWSI1	0.973	46.31			
TWSI2	0.968	45.092			
TWSI3	0.946	—			

Note: ATT = attitude toward the behavior; SN = subjective norms; PBC = perceived behavioral control; EB = emotional bonding; IT = interpersonal trust; TWSI = tourists’ waste sorting intentions.

**Table 3:** Results of discriminant validity.

Construct	ATT	SN	PBC	EB	IT	TWSI
ATT	0.915					
SN	0.335	0.896				
PBC	0.245	0.376	0.881			
EB	0.417	0.410	0.225	0.884		
IT	0.209	0.303	0.111	0.159	0.780	
TWSI	0.372	0.328	0.253	0.239	0.276	0.962

Note: ATT = attitude toward the behavior; SN = subjective norms; PBC = perceived behavioral control; EB = emotional bonding; IT = interpersonal trust; TWSI = tourists’ waste sorting intentions.

**Table 4:** Structural model assessment and hypothesis test outcomes.

Hypotheses	Path	Standardized Coefficient	*t*-Value	Results
H1	ATT→TWSI	0.252	4.687 ***	Supported
H2	SN→TWSI	0.141	2.436 *	Supported
H3	SN→ATT	0.129	2.223 *	Supported
H4	PBC→TWSI	0.115	2.197 *	Supported
H5	PBC→ATT	0.112	2.145 *	Supported
H6	EB→TWSI	0.024	0.441	Not Supported
H7	EB→ATT	0.322	6.088 ***	Supported
H8	IT→TWSI	0.164	3.061 **	Supported
H9	IT→ATT	0.106	2.011	Supported

Note: * *p* < 0.05, ** *p* < 0.01, *** *p* < 0.001. ATT = attitude toward the behavior; SN = subjective norms; PBC = perceived behavioral control; EB = emotional bonding; IT = interpersonal trust; TWSI = tourists’ waste sorting intentions.

**Table 5:** Specific mediation test results.

Mediating Hypothesized Path	Indirect Effects	95% Bias-Corrected Confidence Intervals			95% Percentile Confidence Intervals			Results
		Lower	Upper	*p*-Value	Lower	Upper	*p*-Value	
SN→ATT→TWSI	0.033	0.005	0.072	0.021	0.003	0.068	0.034	Supported
PBC→ATT→TWSI	0.028	0.005	0.062	0.017	0.002	0.059	0.030	Supported
EB→ATT→TWSI	0.081	0.043	0.130	0.000	0.041	0.127	0.001	Supported
IT→ATT→TWSI	0.027	0.005	0.056	0.017	0.004	0.054	0.023	Supported

Note: ATT = attitude toward the behavior; SN = subjective norms; PBC = perceived behavioral control; EB = emotional bonding; IT = interpersonal trust; TWSI = tourists’ waste sorting intentions.

**Table 6:** Test results of the model comparison between the TPB and social capital.

Model Category	R^2^: ATT	R^2^: TWSI
M0: TPB	0.129	0.195
M1: Social capital	—	0.153
M2: M0 + M1	0.227	0.220

Note: ATT = attitude toward the behavior; TWSI = tourists’ waste sorting intentions.

## Data Availability

Not applicable.
